# RETRACTION: 5′‐Adenosine Monophosphate‐Induced Hypothermia Attenuates Brain Ischemia/Reperfusion Injury in a Rat Model by Inhibiting the Inflammatory Response

**DOI:** 10.1155/mi/9868650

**Published:** 2026-03-25

**Authors:** Mediators of Inflammation

RETRACTION: Y.‐F. Miao, H. Wu, S.‐F. Yang, J. Dai, Y.‐M. Qiu, Z.‐Y. Tao, and X.‐H. Zhang, “5′‐Adenosine Monophosphate‐Induced Hypothermia Attenuates Brain Ischemia/Reperfusion Injury in a Rat Model by Inhibiting the Inflammatory Response,” *Mediators of Inflammation*, 2015, 520745, https://doi.org/10.1155/2015/520745.

The above article, published online on 22 March 2015 in Wiley Online Library (wileyonlinelibrary.com), has been retracted by agreement between the journal’s Chief Editor, Anshu Agrawal; and John Wiley & Sons Ltd. A third party reported on PubPeer [1] that images in Figure 4 in this article had been published in another article by some of the same authors [Hu et al. 2018 (https://doi.org/10.3892/mmr.2017.7858)]. Additional investigation by the publisher found evidence of image overlap in Figure 4, image duplication and rotation of Figure 4B in another article by some of the same authors [Miao et al. 2018 (https://doi.org/10.1038/s41598-017-18914-6)], and that the beta‐actin band in Figure 6 had been duplicated from an earlier article by some of the same authors [Ding et al. 2012 (https://doi.org/10.3390/ijms13056089)].

The authors responded to an inquiry by the publisher and provided original data as well as further supplementary material to validate the experimental results in the article. However, a review of the provided materials found that the data could not be verified when compared to the results published in the article.

The retraction has been agreed to because the evidence of image duplication fundamentally compromises the editors’ confidence in the results presented. The authors did not respond to the notice regarding the retraction.
